# A Unified Description of Salt Effects on the Liquid–Liquid Phase Separation of Proteins

**DOI:** 10.1021/acscentsci.3c01372

**Published:** 2024-02-08

**Authors:** Chao Duan, Rui Wang

**Affiliations:** † Department of Chemical and Biomolecular Engineering, University of California Berkeley, Berkeley, California 94720, United States; ‡ Materials Sciences Division, Lawrence Berkeley National Lab, Berkeley, California 94720, United States

## Abstract

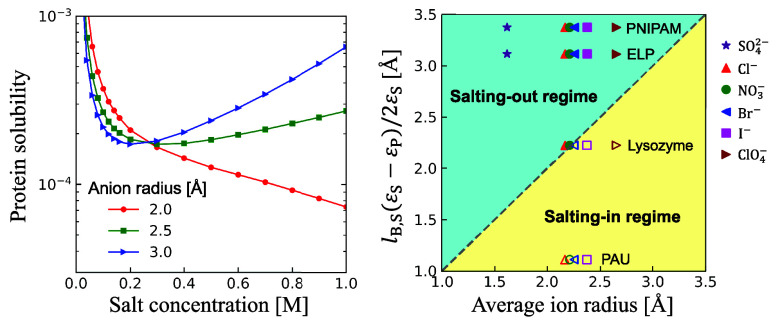

Protein aggregation via liquid–liquid phase separation (LLPS) is ubiquitous in nature and is intimately connected to many human diseases. Although it is widely known that the addition of salt has crucial impacts on the LLPS of proteins, full understanding of the salt effects remains an outstanding challenge. Here, we develop a molecular theory that systematically incorporates the self-consistent field theory for charged macromolecules into the solution thermodynamics. The electrostatic interaction, hydrophobicity, ion solvation, and translational entropy are included in a unified framework. Our theory fully captures the long-standing puzzles of the nonmonotonic salt concentration dependence and the specific ion effect. We find that proteins show salting-out at low salt concentrations due to ionic screening. The solubility follows the inverse Hofmeister series. In the high salt concentration regime, protein continues salting-out for small ions but turns to salting-in for larger ions, accompanied by the reversal of the Hofmeister series. We reveal that the solubility at high salt concentrations is determined by the competition between the solvation energy and translational entropy of the ion. Furthermore, we derive an analytical criterion for determining the boundary between the salting-in and salting-out regimes, which is in good agreement with experimental results for various proteins and salt ions.

## Abstract 2

This work uses a simple theory to capture the long-standing puzzles of the nonmonotonic salt concentration dependence and the specific ion effect in the liquid−liquid phase separation of proteins.

## 1 Introduction

Protein aggregation is ubiquitous in living cells, through which plenty of biomolecular condensates can be assembled.^1,2^ These biomolecular condensates play a vital role in cellular organization and functions, such as the formation of nucleoli,^3^ heterochromatin and ribonucleoprotein granules^4,5^ as well as signal transduction within the cytoplasm.^6−8^ In addition, the aggregation of various misfolded proteins is intimately linked to many neurodegenerative diseases including Alzheimer’s, Parkinson’s, diabetes, and prion diseases.^9,10^ Evidence is mounting that protein aggregation proceeds via a liquid–liquid phase separation (LLPS), which is manifested as the formation of a dense phase often resembling liquid droplets and a coexisting dilute phase.^11−14^ Revealing the essential physical chemistry of the LLPS-driven aggregation will help delineate the functions of biomolecular condensates and provides useful guidance for the therapy of diseases.^15−17^ In spite of increasing academic interests, understanding and regulating LLPS of protein remains a big challenge.^18^

The salt effect on LLPS of protein is one of the most long-standing puzzles. It is well-known that the ionic environment has critical impacts on the LLPS; besides, the addition of salt also provides an effective tool to modulate it.^19^ However, this salt effect is very complicated: the LLPS of protein has nontrivial dependence on both the salt concentration and the chemical identity of ions (usually known as the specific ion effect or Hofmeister series effect).^20−24^ Zhang and Cremer measured the cloud point of positively charged lysozyme solutions.^25^ At low salt concentrations, they found that the solubility of lysozyme decreases as salt concentration increases, i.e., protein salting-out. The increase of solubility follows the inverse Hofmeister series of anion. In contrast, at high salt concentrations, lysozyme continues to show salting-out for some anions (e.g., Cl^–^), whereas other anions (e.g., Br^–^ and I^–^) enhance the lysozyme solubility, i.e., protein salting-in. The solubility increase follows the direct Hofmeister series in the high salt concentration regime. Neither the nonmonotonic salt concentration effect nor the specific ion effect can be explained, even qualitatively, by the standard mean-field Poisson–Boltzmann (PB) theory.^26^ Similar salt-dependent behaviors have also been observed in other protein solutions^27−30^ and soft matter systems such as synthetic polymers^31−33^ and colloidal dispersions,^34,35^ implying the universality of the salt effects on LLPS.

Many theoretical and computational efforts have been made to explain these salt effects.^25,36−42^ Kastelic et al. assumed a phenomenological model for the interaction energy between proteins, where the well depths in the presence of different alkali–halide salts were fitted to experimental data.^36^ They suggested that the salt effect on LLPS is mainly attributed to the ionic screening, but the salting-in behavior and the reversal of Hofmeister series observed at high salt concentrations have not been captured. Zhang and Cremer developed a modified binding isotherm model.^25^ The model parameters representing the effectiveness and equilibrium constant for the association of a specific anion to the protein surface were fitted to the measured cloud point. They found that the salt effect in the high salt concentration regime is correlated to the interfacial tension of protein surrounded by anions with different polarizability. Furthermore, using a modified PB theory to account for ion size and polarizability, Boström et al. suggested that the reversal of the Hofmeister series at high salt concentrations originates from the inversion of effective surface charge of proteins.^37^ However, there has been no theory to date that can unify the description of the salt effects on the LLPS of proteins for the entire salt concentration regime. The underlying physical chemistry, particularly for the counterintuitive behaviors observed at high salt concentrations, is still unclear.

To uncover the salt effect on LLPS of protein, we develop a molecular theory which systematically includes the electrostatics, hydrophobic interaction, ion solvation, and translational entropy of protein in a unified framework. Compared with the existing theories, we have made the following two major improvements. First, we explicitly account for the highly localized density fluctuation of proteins in the dilute phase rather than assuming random mixing as invoked in the Flory–Huggins (F–H) theory.^43,44^ This enables the accurate treatment of the ionic screening effect on a charged protein aggregate. Second, we include the self-energy of ions as a result of electrostatic fluctuation, which captures the salt effects beyond the mean-field PB level.^45,46^ Our theory predicts that protein salting-out at low salt concentrations is attributed to the screening effect, whereas protein solubility at high salt concentrations is determined by the competition between the solvation energy and translational entropy of ions. Furthermore, we derive an analytical criterion for determining the boundary between the salting-in and salting-out regimes for different proteins and ions. The theoretical prediction is in good agreement with experimental data reported in literature.

## 2 Theory

**Figure 1:**
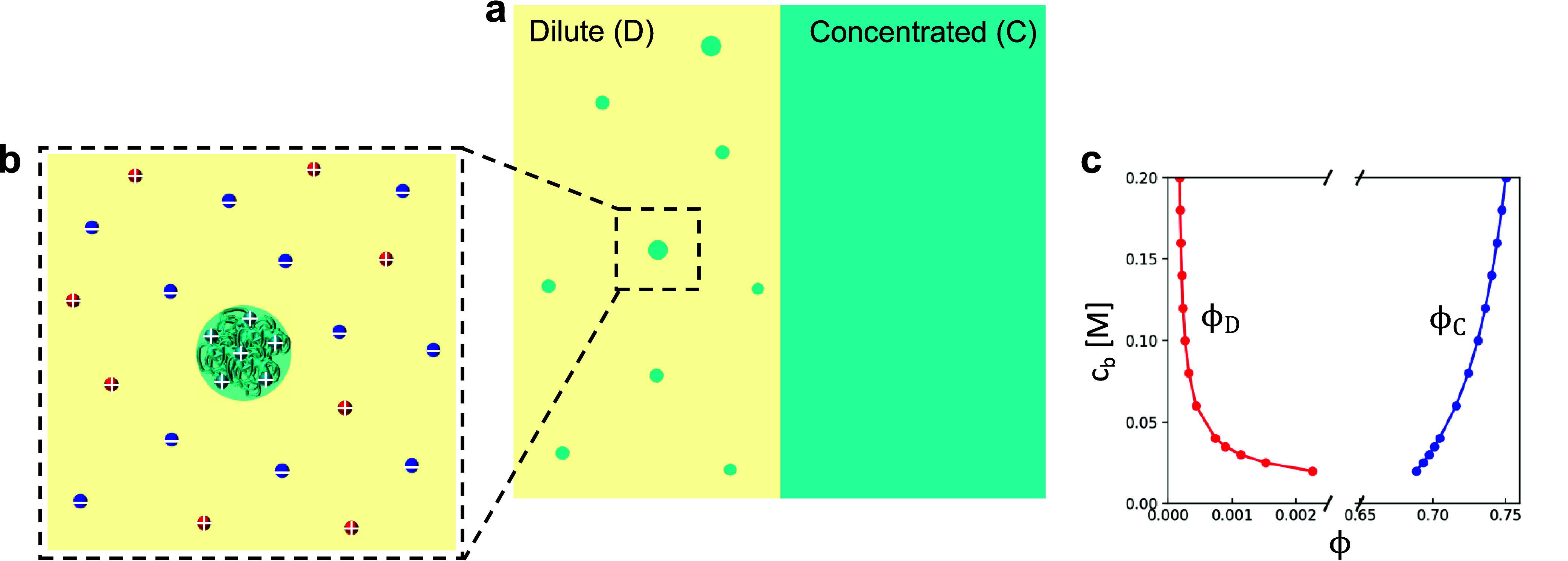
(a) Schematic of the total system consisting of coexisting dilute phase (D) and concentrated phase (C). The dilute phase is an assemble of protein aggregates with different aggregation numbers. (b) A subsystem containing one isolated aggregate in the presence of salt ions. (c) A representative phase diagram plotting the equilibrium volume fractions of the two coexisting phases (ϕ_D_ and ϕ_C_) as a function of bulk salt concentration *c*_b_. *a*_+_ = *a*_–_ = 2.5 Å, *z*_+_ = *z*_–_ = 1, ε_P_ = 30, and ε_S_ = 80.

The solubility of protein in a salt solution is built upon the equilibrium between a dilute phase and a protein-rich concentrated phase, as illustrated in Figure 1a. The concentrated solution can be modeled by a homogeneous liquid-like condensate due to the negligible density fluctuation and the surface contribution. However, the description of the dilute phase is nontrivial because of the large localized density fluctuation. An instantaneous picture of the dilute protein solution has localized high concentrations where the proteins are located and pure salt solutions elsewhere. This is an exactly different scenario compared to that envisioned in the random mixing picture of F–H theory used in existing work.^15−17,47,48^ To account for this large localized density fluctuation in the dilute phase, we focus on the subvolume of the entire solution containing only one isolated protein or one multiprotein aggregate (see Figure 1b). The density profile and free energy of the protein/aggregate is obtained by applying the self-consistent field theory (SCFT) in the subvolume. This information is then incorporated into the framework of dilute solution thermodynamics to reconstruct the solution behavior of the entire dilute phase.

### 2.1 Self-Consistent Field Theory for an Isolated Protein/Aggregate

As shown in Figure 1b, we consider a subvolume consisting of an isolated aggregate of *m* proteins and *n*_S_ solvent molecules in the presence of *n*_±_ mobile ions with a valency *z*_±_. The term *m* = 1 specifies the case of an isolated protein. The subvolume is taken as a semicanonical ensemble: the number of proteins is fixed, whereas solvent and mobile ions are connected with a bulk salt solution of ion concentration *c*_±_^*b*^ that maintains the chemical potentials of the solvent μ_S_ and ions μ_±_.^44,49^ The proteins considered here are assumed to be unfolded or intrinsically disordered, where the widely adopted charged macromolecular model is invoked to describe these proteins.^17,50,51^ This model is also general for synthetic polyelectrolytes and other biomacromolecules.^52^ The charged macromolecule is assumed to be a Gaussian chain of *N* Kuhn segments with a Kuhn length *b*. The smeared charge model is adopted to describe the backbone charge distribution with the charge density α.^53^ For simplicity, the volumes of the chain segment and the solvent molecule are assumed to be the same, *v*_0_. The local hydrophobic interaction between the protein and solvent is described by the Flory parameter χ. The key results of the SCFT are the following set of equations for protein density ρ_P_(**r**), conjugate fields ω_P_(**r**) and ω_S_(**r**), electrostatic potential ψ(**r**), and ion concentration *c*_±_(**r**) (see the Supporting Information (SI), Section I for the detailed derivation):
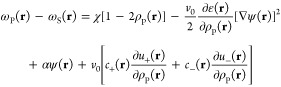
1a
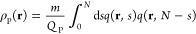
1b
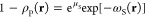
1c
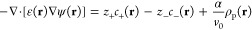
1d
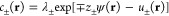
1ewhere ε(**r**) = *kTε*_0_ε_*r*_(**r**)/*e*^2^ is the scaled permittivity with ε_0_ the vacuum permittivity, *e* the elementary charge and ε_*r*_(**r**) the local dielectric constant. ε_*r*_(**r**) can be evaluated based on the local composition.^54,55^ Here a linear mixing rule is adopted which leads to ε_*r*_(**r**) = ε_P_ρ_P_(**r**) + ε_S_(1 – ρ_P_(**r**)), with ε_P_ and ε_S_ the dielectric constant of the pure protein and solvent, respectively.^54−56^ λ_±_=*e*^μ_±_^/*v*_±_ is the fugacity of the ions controlled by the bulk salt concentration. *Q*_P_ is the single-chain partition function given by *Q*_P_ = (1/*v*_0_)*∫*d**r***q*(**r**, *N*), whereas *q*(**r**, *s*) is the chain propagator determined by the diffusion equation
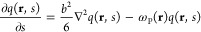
2with *u*_±_(**r**) in eq 1e in the self-energy of ions resulting from the fluctuation of the electrostatic field.^45,46^ If the nonuniversal contribution of the fluctuation in the length scale of the ion size is retained, *u*_±_(**r**) reduces to the local Born energy as
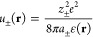
3with *a*_±_ the Born radius of ions. The Born solvation energy accounts for the electrostatic interaction between the ion and the local dielectric medium.^54,57^ It captures the fact that ions are more preferable to be distributed in the medium with a higher dielectric constant. For systems with spatially varying dielectric permittivity, *u*_±_ is not a constant, and cannot be adsorbed into the redefinition of the chemical potential. It will thus affect both the ion distribution and protein density profile, as indicated in eqs 1a and 1e. The nonlocal contributions of electrostatic fluctuation, such as ion correlation and image force, can be rigorously included into the self-energy through Gaussian variational approach.^45,46^ We refer interested readers to the relevant literature for more details. The free energy of the subsystem is then
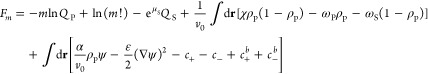
4

### 2.2 Phase Equilibrium

The protein solution in the dilute phase can be reconstructed by incorporating the density profile and free energy of the *m*-aggregate obtained from SCFT into the framework of dilute solution thermodynamics.^44^ The free energy density of the entire dilute solution with volume *V*, including the translational entropy of aggregates, can be written as
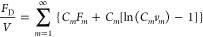
5where *C*_*m*_ is the concentration of the *m*-aggregate, and *v*_*m*_ is a reference volume which, for simplicity, can be taken as the volume of the *m*-aggregate. *C*_*m*_*v*_*m*_ thus becomes the corresponding volume fraction ϕ_*m*_ of the *m*-aggregate. In eq 5, the interaction between different aggregates is ignored under the assumption of a sufficiently dilute solution. The equilibrium concentration of *m*-aggregate can be obtained by minimization of the free energy density in eq 5 subject to fixed total protein concentration ∑_*m* = 1_^*∞*^*mC*_*m*_, which results in the following distribution:
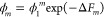
6

Here, Δ*F*_*m*_ = *F*_*m*_ – *mF*_1_ is the free energy of formation of the *m*-aggregate from *m* isolated proteins.

The protein solution in the concentrated phase can be modeled as an infinitely large aggregate with a uniform protein density. The free energy density is directly obtained by applying SCFT to a homogeneous system, and eq 4 becomes
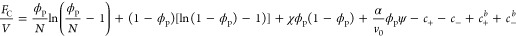
7where ψ is the electrostatic potential difference between the concentrated phase and the dilute phase usually known as Donnan potential or Galvani potential.^46,54^ ψ is obtained by applying the charge neutrality constraint to the homogeneous concentrated phase.

The equilibrium between the protein dilute phase and the protein concentrated phase is determined by the respective equality of the chemical potential of the protein and the solvent in the two coexisting phases, which results in
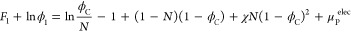
8a
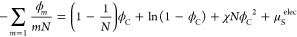
8bwhere ϕ_C_ is the equilibrium volume fraction of protein in the concentrated phase and ϕ_*m*_ is the equilibrium volume fraction of the *m*-aggregate in the dilute phase given by eq 6. The total volume fraction of protein in the dilute phase is thus ϕ_D_ = *∑*_*m*=1_ϕ_*m*_. It should be noted that the sum on the left-hand side of eq 8b is the dimensionless osmotic pressure in dilute phase (in accordance with Van’t Hoff law) as expected for an ideal solution.^58^ μ_P_^elec^ and μ_S_^elec^ are the electrostatic contributions in the chemical potentials of protein and solvent, respectively, which are given by
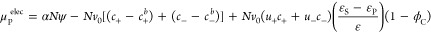
9a
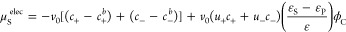
9b

It is worth noting that the three terms on the right-hand side of eq 9a represent the contributions from the energy of a charged protein in the electrostatic field, translational entropy, and the solvation energy of salt ions, respectively. For each salt concentration *c*_*b*_ in the bulk salt solution (i.e., reservoir), the equilibrium volume fractions in the coexisting dilute and concentrated phases ϕ_D_ and ϕ_C_ are obtained by solving eqs 8a and 8b simultaneously, from which the phase diagram as illustrated in Figure 1c can be obtained.

## 3 Results and Discussion

In the current work, we focus on the salt concentration effect and specific ion effect. The number of Kuhn segments in the protein is set as *N* = 50 with *b* = 1.0 nm. We use the simple system of a homogeneous chain with a uniform backbone charge distribution to illustrate the fundamental physical chemistry. The backbone charge density α = +0.05, where positive α is adopted to facilitate the comparison with the corresponding proteins studied in experiments.^25,27,28,30^ The volume of the chain segment and the solvent molecule is assumed to be the same as *v*_0_ = 1.0 nm^3^. The temperature is set to be 298 K with the Flory parameter χ = 1.2. The numerical details are provided in SI, Section II.

### 3.1 Salt Effects on the Protein Solubility

**Figure 2:**
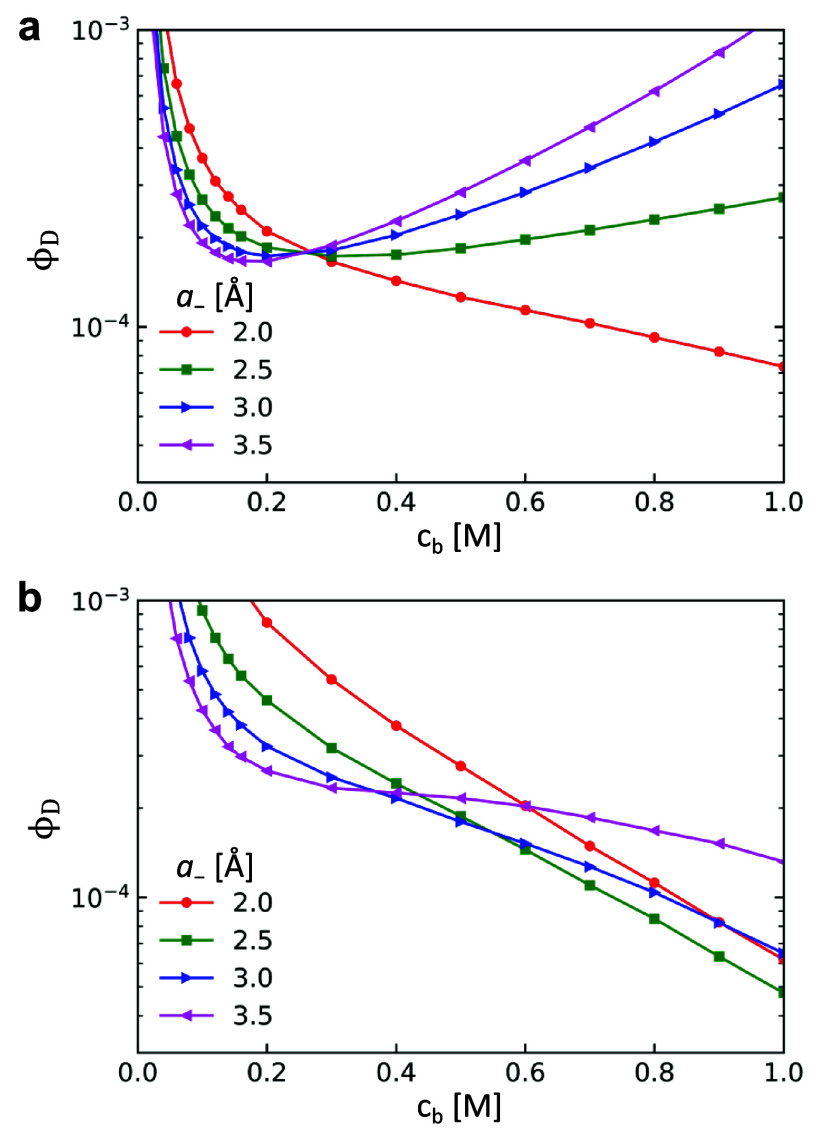
Protein solubility ϕ_*D*_ as a function of salt concentration *c*_*b*_ for anions with different radius *a*_–_. *a*_+_ = 2.5 Å, *z*_+_ = *z*_–_ = 1, ε_S_ = 80. (a) ε_P_ = 30 and (b) ε_P_ = 10.

The salt effects on LLPS of proteins observed in experiments show complicated dependence on both the salt concentration and the chemical identity of ions. We theoretically investigate the protein solubility for different salt concentrations and various anion radii. Here, the solubility is represented by ϕ_D_, the equilibrium volume fraction of the dilute phase on the coexistence curve (see Figure 1c). Figure 2a shows that the solubility decreases as *c*_*b*_ increases in the low salt concentration regime (*c*_*b*_ < 0.2 M), indicating protein salting-out. At the same *c*_*b*_, the solubility decreases with the increase of anion radius, consistent with the trend of the inverse Hofmeister series. In contrast, in the high salt concentration regime (*c*_*b*_ > 0.2 M), protein continues salting-out for small anion (*a*_–_ = 2.0 Å), but turns to salting-in for larger ions. The solubility increases with the increase of the anion radius, indicating the direct Hofmeister series. The dependence of LLPS on both the salt concentration and the specific ions predicted by our theory is in good agreement with the solubility measurements of lysozyme in Zhang and Cremer’s experiments.^25^ Particularly, they found salting-out behavior at high salt concentrations only for small Cl^–^, whereas all other larger anions show salting-in behavior, as exactly captured by Figure 2a.

The salt effects on the solubility in the high salt concentration regime also depend on the property of protein. If a protein with lower dielectric constant (ε_P_ = 10) is adopted as shown in Figure 2b, it exhibits salting-out in the entire salt concentration regime for all the anions with *a*_–_ ≤ 3.5 Å. This is in stark contrast to the behavior predicted for proteins with high ε_P_. It is interesting to note that the same trend has also been reported in experiments. Cho et al. measured the solubility of elastin-like polypeptide which has lower dielectric constant than lysozyme.^27^ All anions investigated in their work show salting-out at high salt concentrations. Similar all-salting-out behavior has been observed by Zhang et al. in the synthetic poly(*N*-isopropylacrylamide) (PNIPAM) system.^31^ The dielectric constant of PNIPAM is less than 5 as reported in the literature.^59^ These experimental results are in good agreement with our theoretical prediction.

As elucidated in eq 8a, the LLPS of the protein is determined by the interplay between the hydrophobic attraction, Coulomb repulsion, and the solvation energy and the translational entropy of ions. The solubility is directly controlled by the effective two-body interaction between proteins: attractive contributions to the interaction favor condensation, whereas repulsive contributions prefer dissolution. The impacts of the aforementioned four contributions on the two-body interaction and their salt-concentration dependence are summarized in Table 1. The hydrophobicity of the protein backbone always leads to effective attraction and is independent of *c*_*b*_, which thus can be neglected when considering salt effects. Coulomb interaction between likely charged proteins is repulsive and decays exponentially with *c*_*b*_ as a result of ionic screening. Furthermore, the contribution of ion solvation is effectively attractive. Ions prefer to be dissolved in the medium with a higher dielectric constant, as indicated by the Born solvation model (eq 3). This selective partition leads to depletion of ions from proteins and thus drives phase separation. Lastly, the translational entropy of ions favors a uniform distribution in the entire solution, which suppresses the aggregation of proteins and thus provides an effective repulsion. As illustrated in eq 9a, the contributions of both the ionic solvation and translational entropy depend linearly on *c*_*b*_. In the following two subsections, we will provide more detailed analysis on the salt effects in the low and high salt concentration regimes, respectively.

**Table 1:** Ingredients of the Effective Two-Body Interaction between Proteins

Contribution	Effective interaction	c_*b*_-dependence
hydrophobicity	attractive	∼ *c*_*b*_^0^
Coulomb interaction	repulsive	∼ *e*^–*κr*^/*r* with κ ∼ *c*_*b*_1/2
ion solvation	attractive	∼ *c*_*b*_
entropy of ion	repulsive	∼ *c*_*b*_

### 3.2 Ionic Screening at Low Salt Concentrations

In the low salt concentration regime, the Coulomb repulsion between proteins dominates compared with the contributions from ionic solvation and translational entropy. Thus, the key factor that determines the salt effects on LLPS is how the Coulomb repulsion is screened by salt ions. The screening effect gets stronger as *c*_*b*_ increases, which leads to the reduction of the effective charge of protein and thus weakens the two-body repulsion. Therefore, the solubility of protein decreases as *c*_*b*_ increases, indicating salting-out behavior (see Figure 2).

**Figure 3:**
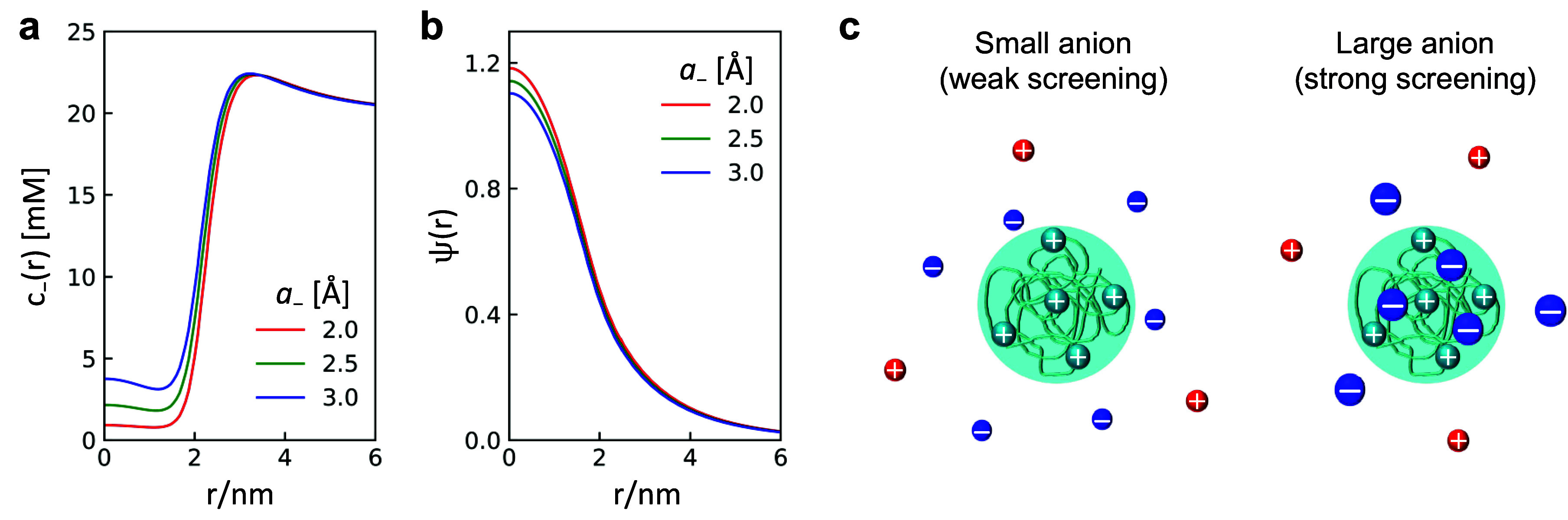
Salt effect on the electrostatic double layer structure around a positively charged protein in the low salt concentration regime. (a) Anion concentration profile *c*_–_(*r*) and (b) electrostatic potential ψ(*r*). *a*_+_ = 2.5 Å, *z*_+_ = *z*_–_ = 1, ε_P_ = 10, ε_S_ = 80, and *c*_*b*_ = 20 mM. (c) Schematics of different screening effects for small anion and large anion.

While the salting-out behavior is universal for all ions in the low salt concentration regime, its degree exhibits a specific ion effect because of the different efficacy of anions in screening the Coulomb repulsion. Based on the Born solvation model, ions are more preferable to be distributed in the solvent region than the protein region as ε_S_ > ε_P_ in most cases. This selective partition is more pronounced for smaller anions. Figure 3 shows the electrostatic double-layer structure around a positively charged protein. Anions with smaller radius are repelled more from the protein center, resulting in a less screened Coulomb potential. Therefore, protein solubility decreases with the increase of the anion radius, in agreement with the trend of inverse Hofmeister series observed in experiments at low salt concentrations. Zhang and Cremer suggested that the specific ion effect on LLPS in the low salt concentration regime is mainly originating from the effectiveness of anions with different sizes in associating with the positively charged protein.^25^ Their explanation is consistent with the mechanism revealed in our results.

### 3.3 Competition between Ion Solvation and Translational Entropy at High Salt Concentrations

**Figure 4:**
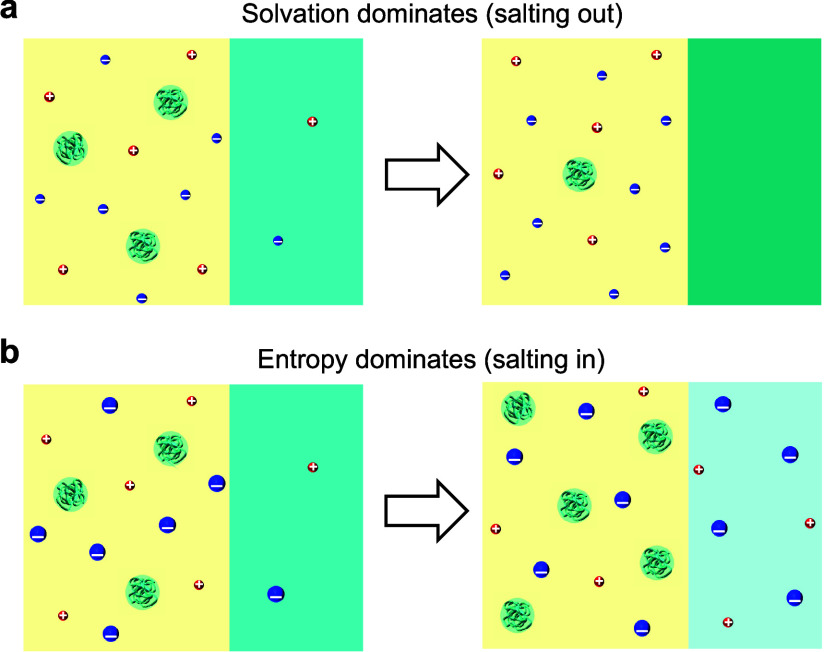
Schematics of salt effects on the LLPS of proteins in the high salt concentration regime. (a) Ion solvation dominates for the case of small ions, which favors salting-out. (b) Translational entropy of ion dominates for the case of large ions, which favors salting-in.

In the high salt concentration regime, the charges carried by proteins are largely screened, and hence, the Coulomb repulsion becomes less significant. The LLPS of protein is mainly determined by the competition between the solvation and translational entropy of ions as illustrated in Figure 4. The tendency for ions to be preferentially solvated by a medium with a higher dielectric constant leads to a driving force for the separation of proteins from the solvent phase. This reduces the solubility, i.e., salting out. On the contrary, the translational entropy of ions favors a uniform distribution in the entire system, which enhances the miscibility between protein and solvent, i.e., salting in.

Based on the electrostatic contributions to the chemical potential in eq 9a, the competition between ion solvation and translational entropy can be quantified by
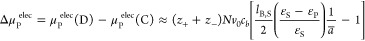
10where *a̅* is the valency-weighted harmonic average radius of cation and anion given by , and *l*_B*,*S_ = *e*^2^/(4*πε*_0_ε_*S*_*kT*) is the Bjerrum length in solvent. The detailed derivation of eq 10 is provided in the SI, Section III. Δμ_P_^elec^ represents the driving force for a single protein to transfer from the concentrated phase (Phase C) to the dilute phase (Phase D). When Δμ_P_^elec^ > 0, ion solvation dominates, and protein prefers to stay in the concentrated phase rather than the dilute phase, which indicates salting-out. When Δμ_P_^elec^ < 0, translational entropy dominates, indicating salting-in. Eq 10 shows that the solvation effect becomes less pronounced as *a̅* increases. This explains our numerical results in Figure 2 and the experimental observations that protein salting-in occurs for larger ions. This can also explain the specific ion effect that protein solubility increases with the anion radius, consistent with the trend of direct Hofmeister series observed in the high salt concentration regime.^25,27,28,30−33^ Furthermore, the solvation energy depends on the ion valency as well. From the expression of *a̅*, ions with higher valency can be equivalently interpreted as monovalent ions with a smaller effective radius. Therefore, multivalent ions promote salting-out. It explains the experimental findings in various protein and polymer solutions that SO_4_^2-^ shows much stronger tendency of salting-out even than Cl^–^, although  is larger than *a*_Cl^–^_.^27,30,31^

As indicated by eq 10, the solubility at high salt concentrations also depends on the dielectric constant of protein ε_P_. Δμ_P_^elec^ decreases with the increase of ε_P_, preferring salting-in. This is consistent with the experimental observation that lysozyme with higher ε_P_ has a stronger tendency of salting-in than elastin-like polypeptide with lower ε_P_. Baldwin measured the solubility of peptide and observed that salting-out becomes more pronounced as the number of hydrocarbon side groups increases.^60^ More hydrocarbon side groups lead to the reduction of the dielectric constant of peptide. Furthermore, Shimada et al. recently investigated the LLPS of ureido-derivatized polymers.^33^ They found that the solubility behavior turns from salting-out to salting-in as more ureido groups are grafted onto the polymer. The ureido group is highly polar and hence expected to increase the dielectric constant of the polymer.^61,62^ Their experimental results can be captured well by our theory.

**Figure 5:**
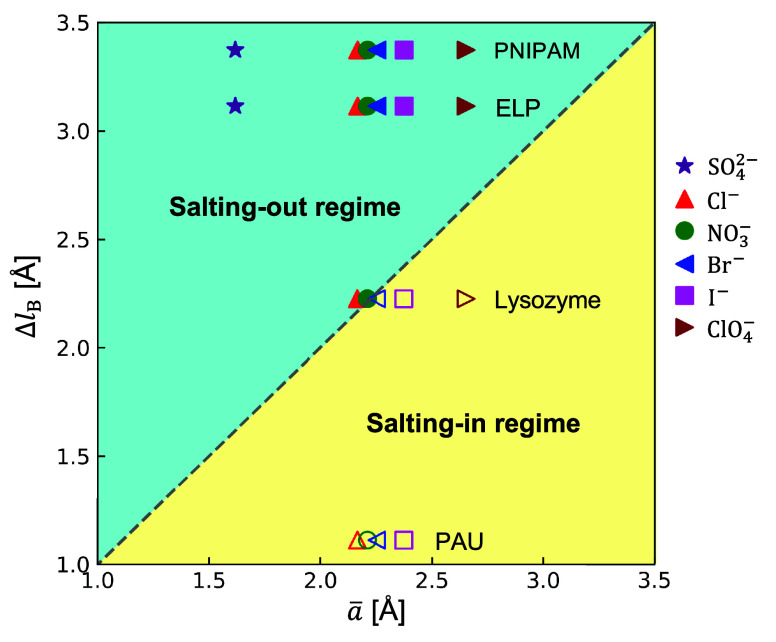
Comparison of the solubility behavior predicted by our theory with experimental results for various proteins and polymers in concentrated sodium solutions with different anions. The dash diagonal line is the universal criterion given by eq 11 for determining the boundary between the salting-in and salting-out regimes. Scattering data points represent the experimental results reported in literature, where open and filled symbols denote salting-in and salting-out behaviors, respectively. The Born radii of Cl^–^, NO_3_^–^, Br^–^, I^–^, ClO_4_^–^, SO_4_^2–^, and Na^+^ are 1.91, 1.98, 2.05, 2.26, 2.83, 3.79, and 2.5 Å, respectively.^64^ The dielectric constant of water ε_S_ = 80. The dielectric constants of lysozyme,^65^ elastin-like polypeptide (ELP),^66^ PNIPAM,^59^ and poly(allylamine)-copoly(allylurea) (PAU)^67^ are 30, 10, 4.2, and 56, respectively.

Our theory provides a simple analytical criterion for determining the solubility behavior, i.e., salting-in versus salting-out. From Δμ_P_^elec^=0 in eq 10, the boundary between the salting-in and salting-out regimes is given by the following universal line:
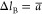
11where Δ*l*_B_ = *l*_B*,*S_(ε_S_ – ε_P_)/2ε_S_ represents a kind of difference in the Bjerrum length between the solvent and protein, which characterizes the solvation preference of the ion in these two media. The relative value between Δ*l*_B_ and *a̅* determines whether ion solvation or translational entropy dominates. This analytical result in eq 11 is confirmed by numerical calculations (Figure S1). To directly compare our theoretical predictions with experimental measurements, Figure 5 shows the solubility behaviors of two proteins (lysozyme^25^ and elastin-like polypeptide^27^) and two synthetic polymers (PNIPAM^31^ and poly(allylamine)-copoly(allylurea)^33^) in solutions of sodium salts with various anions. For a specific pair of protein and anion, the salting-in result observed in the experiment is denoted by an open symbol, whereas the salting-out result is denoted by a filled symbol. These two types of data points are located almost exactly within the corresponding regimes separated by the universal line predicted by eq 11. The protein solubility increases following SO_4_^2–^, Cl^–^, NO_3_^–^, Br^–^, I^–^, and ClO_4_^–^, precisely the direct Hofmeister series.^20,21,63^ It is clear that our theoretical result is quite universal, which captures the known salt concentration effect and specific ion effect on LLPS of different proteins and polymers.

Our theory only needs to invoke parameters such as the valency and radius of ions as well as the dielectric constant of protein, which can either be adopted from the literature or measured in experiments. It is also interesting to note that our theory captures the salt effects on LLPS by only considering the contribution of Born solvation energy in the ion–protein interactions, indicating its dominant role for simple monovalent ions such as halogen anions. However, for ions with more complex constitutions and structures, other contributions such as hydration, dispersion, and polarization should also be taken into account.^68^ Studies have shown that such interactions are the driving forces for many specific ion effects.^35,57,63,64^ Our work suggests that the existence and relative importance of these higher order effects on LLPS can only be evaluated when the essential Born solvation energy and translational entropy of ions are systematically treated as in our theory.

## 4 Conclusions

We develop a self-consistent theory to study salt effects on the LLPS of protein solutions by systematically incorporating electrostatic interaction, hydrophobicity, ion solvation, and transnational entropy into a unified framework. Our theory has made important improvements compared to the previous mean-field work. Both the highly localized density fluctuation of proteins in the dilute phase and the electrostatic fluctuation (manifested by the self-energy of ions) are explicitly accounted for. The long-standing puzzles of the nonmonotonic salt concentration dependence and the specific ion effect are fully captured by our theory. We find that proteins show salting-out at low salt concentrations due to ionic screening. The solubility decreases with the increase in anion radius, following the inverse Hofmeister series. On the other hand, in the high salt concentration regime, protein continues salting-out for small ions but turns to salting-in for larger ions. The Hofmeister series is reversed to the direct sequence. We reveal that both the turning of solubility from salting-out to salting-in and the reversal of the Hofmeister series are attributed to the competition between the solvation energy and translational entropy of ions. Furthermore, we derive an analytical criterion for determining the boundary between the salting-in and salting-out regimes. The theoretical prediction is in good agreement with the experimental results for various proteins and polymers in sodium solutions with a broad range of anions.

Our theory reveals the essential physical chemistry of salt effects on LLPS using a simple charged macromolecular model, which can also be applied to other soft matter systems. The theory can be generalized to macromolecules with more complicated structures (e.g., chain architecture, heterogeneous composition and charge distribution, local rigidity, helicity, etc.) and interactions that better represent real proteins. Although the charged macromolecular model seems to be applicable only to unfolded or intrinsic disordered proteins, the mechanism controlling salting-in versus salting-out elucidated here is applicable to both folded and unfolded proteins. This is because the description of a giant liquid-like condensate is not sensitive to the folding details of a single protein. Furthermore, our theory captures the salt effects on LLPS by only considering the contribution of Born energy in the ion solvation, indicating its dominant role for simple ions such as halogen anions. However, other contributions such as hydration, dispersion, and polarization should also be taken into account for ions with more complex structures. These effects can be straightforwardly incorporated into the theoretical framework. Furthermore, the electrostatic correlations between ions, which becomes more important for multivalent salts, can also be systematically included in our theory using the Gaussian variational approach.^45,46^ The existence and relative importance of these higher order effects on LLPS can only be evaluated when the essential Born energy and translational entropy of ions are accurately treated as in our work. The fundamental insight revealed here provides important guidance for modulating the LLPS of proteins via the addition of salt as an effective tool, which helps us understand the functions of cellular organization and rationally design therapy for diseases.

## Supporting Information Available

Derivation of the self-consistent field theory; details of numerical calculation; quantifying the competition between ion solvation and translational entropy; supplementary figure (PDF)
