# Supplementary material for: A Unified Description of Salt Effects on the Liquid–Liquid Phase Separation of Proteins
Source: ACS Cent Sci. 2024 Feb 8;10(2):460–8. doi: 10.1021/acscentsci.3c01372 (PMC10906038; doi:10.1021/acscentsci.3c01372)
Supplement: Supplementary file 1 — oc3c01372_si_001.pdf [file oc3c01372_si_001.pdf]

Supporting Information for: A Unified Description of Salt Effects on the Liquid-Liquid Phase Separation of Proteins

Chao Duan[†] and Rui Wang[*,†,‡]

†*Department of Chemical and Biomolecular Engineering, University of California Berkeley, Berkeley, California 94720, United States*

‡*Materials Sciences Division, Lawrence Berkeley National Lab, Berkeley, California 94720, United States*

E-mail: ruiwang325@berkeley.edu

Contents

I. Derivation of the self-consistent field theory S2

II. Details of numerical calculation S7

III. Quantifying the competition between ion solvation and translational entropy S8

IV. Supplementary figure S10

I. Derivation of the self-consistent field theory

In this section, we provide detailed derivation of the key equations of our theory (Eqs. (1)-(4) in the main text). The semicanonical partition function can be written as

$$\Xi = \frac{1}{m! v_P^{Nm}} \sum_{n_\gamma=0}^{\infty} \prod_{\gamma} \frac{e^{\mu_\gamma n_\gamma}}{n_\gamma! v_\gamma^{n_\gamma}} \prod_{i=1}^{m} \int \hat{D}\{\mathbf{R}_i\} \prod_{j=1}^{n_\gamma} \int \mathrm{d}\mathbf{r}_{\gamma,j} \prod_{\mathbf{r}} \delta\left[\hat{\rho}_P(\mathbf{r}) + \hat{\rho}_S(\mathbf{r}) - 1\right] \exp(-H) \tag{S1}$$

where $\gamma = s, \pm$ represents all the small molecules in the system. v_P and v_γ are the volume of the chain segments and small molecules, respectively. For simplicity, we assume $v_P = v_S = v_0$. $\int \hat{D}\{\mathbf{R}_i\}$ denotes integration over all chain configurations weighted by the Gaussian-chain statistics. $\hat{\rho}_P(\mathbf{r})$ and $\hat{\rho}_S(\mathbf{r})$ are the local instantaneous volume fraction of protein and solvent, respectively. The δ functional accounts for the incompressibility. The Hamiltonian H in Eq. (S1) is given by

$$H = \frac{\chi}{v_0} \int \mathrm{d}\mathbf{r} \hat{\rho}_P(\mathbf{r}) \hat{\rho}_S(\mathbf{r}) + \frac{1}{2} \int \mathrm{d}\mathbf{r} \int \mathrm{d}\mathbf{r}' \hat{c}_e(\mathbf{r}) C(\mathbf{r}, \mathbf{r}') \hat{c}_e(\mathbf{r}') \tag{S2}$$

The transfer from particle-based to field-based representation is achieved through the identity transformation as:

$$1 = \int D\rho_k \prod_{\mathbf{r}} \delta[\rho_k(\mathbf{r}) - \hat{\rho}_k(\mathbf{r})] = \int D\rho_k D\omega_k \exp\left\{ i \int d\mathbf{r} \omega_k(\mathbf{r}) [\rho_k(\mathbf{r}) - \hat{\rho}_k(\mathbf{r})] \right\} \quad (k = P, S) \tag{S3}$$

where the right hand side of the equation arises from the Fourier representation of the δ-function with $\omega_i(\mathbf{r})$ being the Fourier conjugate field to $\rho_i(\mathbf{r})$. To further decouple the interactions between charged particles, Hubbard-Stratonovich transform is performed for

the total charge density $c_e(\mathbf{r})$, which introduces the conjugate field $\psi(\mathbf{r})$

$$\exp\left\{-\frac{\beta e^2}{2}\int \mathrm{d}\mathbf{r}\int \mathrm{d}\mathbf{r}'\hat{c}_e(\mathbf{r})C(\mathbf{r},\mathbf{r}')\hat{c}_e(\mathbf{r}')\right\} =$$
$$\mathcal{N}_\psi\int D\psi\exp\left\{-\frac{\beta}{2}\int\mathrm{d}\mathbf{r}\int\mathrm{d}\mathbf{r}'\psi_c(\mathbf{r})C^{-1}(\mathbf{r},\mathbf{r}')\psi_c(\mathbf{r}') - i\int\mathrm{d}\mathbf{r}\hat{c}_e(\mathbf{r})\beta e\psi(\mathbf{r})\right\} \tag{S4}$$

Where $\mathcal{N}_\psi^{-1} = \int D\psi\exp\{-(\beta/2)\int\mathrm{d}\mathbf{r}\int\mathrm{d}\mathbf{r}'\psi_c(\mathbf{r})C^{-1}(\mathbf{r},\mathbf{r}')\psi_c(\mathbf{r}')\}$ is the normalization constant. $C^{-1}(\mathbf{r},\mathbf{r}') = -\nabla\cdot[\epsilon(\mathbf{r})\nabla]\delta(\mathbf{r}-\mathbf{r}')$ is the inverse of Coulomb operator $C(\mathbf{r},\mathbf{r}')$. To economize notation, we scale the quantities $\beta e\psi(\mathbf{r})$ and $\epsilon(\mathbf{r})/(\beta e^2)$ into $\psi(\mathbf{r})$ and $\epsilon(\mathbf{r})$, respectively, and Eq. (S2) can be rewritten as

$$\exp\left\{-\frac{\beta}{2}\int \mathrm{d}\mathbf{r}\int \mathrm{d}\mathbf{r}'\hat{c}_e(\mathbf{r})C(\mathbf{r},\mathbf{r}')\hat{c}_e(\mathbf{r}')\right\} =$$
$$\mathcal{N}_\psi\int D\psi\exp\left\{\int\mathrm{d}\mathbf{r}\left[\frac{\epsilon(\mathbf{r})}{2}(i\psi(\mathbf{r}))^2 - \left(z_+\hat{c}_+(\mathbf{r}) - z_-\hat{c}_-(\mathbf{r}) + \frac{\alpha}{v_0}\rho_P(\mathbf{r})\right)i\psi(\mathbf{r})\right]\right\} \tag{S5}$$

The partition function Ξ in Eq. (S1) can then be cast into the following functional integral

$$\begin{aligned}\Xi = &\int D\rho_P D\rho_S D\omega_P D\omega_S D\psi D\eta\\ &\cdot\frac{1}{m!v_0^{Nm}}\sum_{n_\gamma=0}^{\infty}\prod_{\gamma}\frac{e^{\mu_\gamma n_\gamma}}{n_\gamma! v_\gamma^{n_\gamma}}\prod_{j=1}^{n_\gamma}\int\mathrm{d}\mathbf{r}_{\gamma,j}\int\prod_{i=1}^{m}D\{\mathbf{R}_i\}\exp\left\{-\sum_{i=1}^{m}\frac{3}{2b^2}\int_0^N\mathrm{d}s\left[\frac{\partial\mathbf{R}_i(s)}{\partial s}\right]^2\right\}\\ &\cdot\exp\left\{-\frac{\chi}{v_0}\int\mathrm{d}\mathbf{r}\rho_P(\mathbf{r})\rho_S(\mathbf{r}) + \int\mathrm{d}\mathbf{r}\left[\frac{\epsilon(\mathbf{r})}{2}|i\psi(\mathbf{r})|^2 - \left(z_+\hat{c}_+(\mathbf{r}) - z_-\hat{c}_-(\mathbf{r}) + \frac{\alpha}{v_0}\rho_P(\mathbf{r})\right)i\psi(\mathbf{r})\right]\right\}\\ &\cdot\exp\left\{\frac{1}{v_0}\int\mathrm{d}\mathbf{r}\left[i\omega_P(\mathbf{r})\left(\rho_P(\mathbf{r})-\hat{\rho}_P(\mathbf{r})\right) + i\omega_S(\mathbf{r})\left(\rho_S(\mathbf{r})-\hat{\rho}_S(\mathbf{r})\right) + i\eta(\mathbf{r})\left(\rho_P(\mathbf{r})+\rho_S(\mathbf{r})-1\right)\right]\right\}\end{aligned} \tag{S6}$$

Note that we omit all the normalization factors and assume $v_P = v_S = v_0$. The Gaussian integral

$$\prod_{i=1}^{m}\int\hat{D}\{\mathbf{R}_i\} = \int\prod_{i=1}^{m}D\{\mathbf{R}_i\}\exp\left\{-\sum_{i=1}^{m}\frac{3}{2b^2}\int_0^N\mathrm{d}s\left[\frac{\partial\mathbf{R}_i(s)}{\partial s}\right]^2\right\}$$

is inserted as well as the Fourier representation of the incompressibility condition

$$\prod_{\mathbf{r}} \delta[\rho_P(\mathbf{r}) + \rho_S(\mathbf{r}) - 1] = \int D\eta \exp\left\{ i \int d\mathbf{r}\eta(\mathbf{r})[\rho_P(\mathbf{r}) + \rho_S(\mathbf{r}) - 1] \right\}$$

Then Eq. (S6) is given by

$$\begin{aligned}\Xi = & \int D\rho_P D\rho_S D\omega_P D\omega_S D\psi D\eta \exp(e^{\beta\mu_S} Q_S) \frac{Q_P^m}{m!} \\ & \cdot \exp\left\{ \int \mathrm{d}\mathbf{r} \left[\lambda_+ e^{-z_+ i\psi(\mathrm{r})} + \lambda_- e^{z_- i\psi(\mathrm{r})} + \frac{\epsilon(\mathbf{r})}{2} |i\psi(\mathbf{r})|^2 + \frac{\alpha}{v_0} \rho_P(\mathbf{r}) i\psi(\mathbf{r}) \right] \right\} \\ & \cdot \exp\left\{ \frac{1}{v_0} \int \mathrm{d}\mathbf{r} \left[-\chi\rho_P(\mathbf{r})\rho_S(\mathbf{r}) + i\omega_P(\mathbf{r})\rho_P(\mathbf{r}) + i\omega_S(\mathbf{r})\rho_S(\mathbf{r}) + i\eta(\mathbf{r}) \left(\rho_P(\mathbf{r}) + \rho_S(\mathbf{r}) - 1 \right) \right] \right\}\end{aligned} \tag{S7}$$

Where $\lambda_\pm = e^{\mu_\pm}/v_\pm$ is the fugacity of the ions.

$$Q_S = \frac{1}{v_0} \int \mathrm{d}\mathbf{r} \exp[-i\omega_S(\mathbf{r})] \tag{S8}$$

is the partition function of solvents.

$$Q_P = \frac{1}{v_0^N} \int D\mathbf{R} \exp\left\{ -\int_0^N \mathrm{d}s \left[\frac{3}{2b^2} \left(\frac{\partial \mathbf{R}(s)}{\partial s} \right)^2 + i\omega_P(\mathbf{R}(s)) \right] \right\} \tag{S9}$$

is the single-chain partition function of PEs. Eq. (S9) can be reexpressed in terms of the chain propagator $q(\mathbf{r}, N)$,[S1]

$$Q_P = \frac{1}{v_0} \int \mathrm{d}\mathbf{r} q(\mathbf{r}, N) \tag{S10}$$

where propagator $q(\mathbf{r}, s)$ satisfies the following modified diffusion equation

$$\frac{\partial q(\mathbf{r}, s)}{\partial s} = \frac{b^2}{6} \nabla^2 q(\mathbf{r}, s) - i\omega_P(\mathbf{r}) q(\mathbf{r}, s) \tag{S11}$$

After functional minimization of Ξ in Eq. (S7) with respect to all the fields $\rho_{p,s}$, $\omega_{p,s}$, η, and ψ (saddle-point approximation) together with the assumption that $\epsilon(\mathbf{r})$ can be expressed as a function of $\rho_P(\mathbf{r})$, we can obtain the following coupled self-consistent equations:

$$\omega_P(\mathbf{r}) = \chi\rho_S(\mathbf{r}) - \eta(\mathbf{r}) - \frac{v_0}{2}\frac{\partial\epsilon(\mathbf{r})}{\partial\rho_P(\mathbf{r})}[\nabla\psi(\mathbf{r})]^2 + \alpha\psi(\mathbf{r}) \tag{S12a}$$

$$\omega_S(\mathbf{r}) = \chi\rho_P(\mathbf{r}) - \eta(\mathbf{r}) \tag{S12b}$$

$$\rho_P(\mathbf{r}) = \frac{m}{Q_P}\int_0^N \mathrm{d}s q(\mathbf{r}, s)q(\mathbf{r}, N - s) \tag{S12c}$$

$$\rho_S(\mathbf{r}) = e^{\mu_S}\exp[-\omega_S(\mathbf{r})] \tag{S12d}$$

$$0 = \rho_P(\mathbf{r}) + \rho_S(\mathbf{r}) - 1 \tag{S12e}$$

$$-\nabla\cdot[\epsilon(\mathbf{r})\nabla\psi(\mathbf{r})] = z_+c_+(\mathbf{r}) - z_-c_-(\mathbf{r}) + \frac{\alpha}{v_0}\rho_P(\mathbf{r}) \tag{S12f}$$

$$c_\pm(\mathbf{r}) = \lambda_\pm\exp[\mp z_\pm\psi(\mathbf{r})] \tag{S12g}$$

In these Eqs. (S12a)$-$(S12g), we have replaced $i\rho_{p,s}$, $i\omega_{p,s}$, $i\eta$, $i\psi$ by $\rho_{p,s}$, $\omega_{p,s}$, η, ψ in anticipation of the fact that the saddle point values of the original fields are purely imaginary. The same replacement will also be done for Eqs. (S8), (S11). Then the equilibrium semicanonical free energy can be obtained as:

$$\begin{aligned} F_m = &-m\log Q_P + \log(m!) - e^{\beta\mu_S}Q_S + \frac{1}{v_0}\int \mathrm{d}\mathbf{r}\,[\chi\rho_P(\mathbf{r})\rho_S(\mathbf{r}) - \omega_P(\mathbf{r})\rho_P(\mathbf{r}) - \omega_S(\mathbf{r})\rho_S(\mathbf{r})] \\ &+ \int \mathrm{d}\mathbf{r}\left\{\frac{\alpha}{v_0}\rho_P(\mathbf{r})\psi(\mathbf{r}) - \frac{\epsilon(\mathbf{r})}{2}[\nabla\psi(\mathbf{r})]^2 - c_+(\mathbf{r}) - c_-(\mathbf{r}) + c_+^b + c_-^b\right\} \end{aligned} \tag{S13}$$

Equations (S12a)-(S12g) are derived in the mean-field framework which cannot describe the effects of spatial varying dielectric medium and the ion-ion correlation as a consequence of the fluctuation of the electrostatic field. To capture the local fluctuation effect, the Born solvation energy $u_\pm(\mathbf{r})$ can be included into the Boltzmann factor in Eq. (S12g) as:

$$c_\pm(\mathbf{r}) = \lambda_\pm\exp[\mp z_\pm\psi(\mathbf{r}) - u_\pm(\mathbf{r})] \tag{S14}$$

where $u_{\pm}(\mathbf{r}) = z_{\pm}^2 / \left[8\pi a_{\pm}\epsilon(\mathbf{r})\right]$ with $a_{\pm}$ the Born radius of cations and anions, respectively. The inclusion of the Born solvation energy can be rigourously achieved by taking the Gaussian fluctuation of the electrostatic field and retaining the nonuniversal contribution in the length scale of the ion size. Equation (S12f) combined with Eq. (S14) is known as the Born-energy augmented Poisson-Boltzmann equation.[S2] Accordingly, Eq. (S12a) can be modified as

$$\begin{aligned}\omega_P(\mathbf{r}) = \chi\rho_S(\mathbf{r}) - \eta(\mathbf{r}) - \frac{v_0}{2}\frac{\partial\epsilon(\mathbf{r})}{\partial\rho_P(\mathbf{r})}[\nabla\psi(\mathbf{r})]^2 + \alpha\psi(\mathbf{r}) \\ + v_0\left[c_+(\mathbf{r})\frac{\partial u_+(\mathbf{r})}{\partial\rho_P(\mathbf{r})} + c_-(\mathbf{r})\frac{\partial u_-(\mathbf{r})}{\partial\rho_P(\mathbf{r})}\right]\end{aligned} \quad \text{(S15)}$$

II. Details of numerical calculation

In this section, numerical details of solving the self-consistent field equations are provided. Based on the symmetry of spherical aggregate, we use spherical coordinate in the numerical calculation. Both the protein density and electrostatic potential field are set to be zero at the boundary of the spherical simulation box. The Crank–Nicolson method is used to solve the modified diffusion equation of chain propagator (eq S11).[S3] The number of points that the chain contour has been discretized is set to be $N_s = 1000$. The grid lattices are set such that the lattice spacings are smaller than $0.1b$. μ_S is set to be -1, such that the free energy of the reservoir of pure salt solution outside of the subvolume is 0. The equilibrium structure and the free energy can be obtained by solving eqs S11-S15 iteratively until convergence. The calculation is accurate if the chosen box is large enough such that both the density profile and electrostatic potential profile reach a plateau at the boundary. We have double checked our calculation to guarantee the achievement of this plateau. We have also ensured that the density profile and free energy of the subvolume does not change if we further enlarge the box size. To accelerate the convergence, we use the following strategy to update the fields. Fields conjugate to the density of protein and solvent molecules are updated by a simple mixing rule, i. e., $\omega_{P,S}^{new} \leftarrow \lambda\omega_{P,S}^{new} + (1-\lambda)\omega_{P,S}^{old}$. The same rule is adopted for updating electrostatic potential ψ and Born energy $u_{\pm}$. The field conjugated to the incompressibility condition is updated by $\eta^{new} \leftarrow \eta^{old} + \kappa(\rho_P + \rho_S - 1)$, where the second term on the r.h.s is adopted to reinforce the incompressibility. λ=0.01 and κ=2.0 are chosen in our calculation. In the iteration process, we choose different initial seeds to confirm that the iteration is converged. The relative errors for the free energy and the incompressibility condition are set to be below 10^{-11} and 10^{-7}, respectively.

III. Quantifying the competition between ion solvation and translational entropy

In this section, we provide detailed derivation of Eq. (10) in the main text, which quantifies the competition between the ion solvation and translational entropy at high salt concentrations. We first consider the case of protein concentrated phase (Phase C). In the high salt concentration regime, the charges carried by proteins are largely screened, hence the contribution from the energy of a charged protein in the electrostatic field to the chemical potential of protein (Eq. (9a)) is neglected. Then the electrostatic chemical potential of protein in Phase C can be approximated as

$$\mu_P^{elec}(C) \approx -Nv_0[(c_+ - c_+^b) + (c_- - c_-^b)] + Nv_0(u_+c_+ + u_-c_-)\left(\frac{\epsilon_S - \epsilon_P}{\epsilon}\right)(1 - \phi_C) \quad \text{(S16)}$$

In Phase C, protein density is very high ($\phi_C \approx 1$) such that the second term in Eq. (S16) can be neglected. In addition, ion concentrations in protein domain are very low ($c_\pm \approx 0$) because ion solvation energy dominates for Phase C and proteins usually have low dielectric constant. Therefore, Eq. (S16) can be finally simplified to

$$\mu_P^{elec}(C) \approx Nv_0(c_+^b + c_-^b) \quad \text{(S17)}$$

On the other hand, in the protein dilute phase (Phase D), the form of Eq. (S16) remains to be valid for the electrostatic chemical potential of protein if protein concentration is very low ($\phi_D \approx 0$) and Phase D can be treated as a homogeneous medium. Ion concentrations in Phase D are taken to be their bulk values ($c_\pm \approx c_\pm^b$). The dielectric constant of the dilute phase and the Born energy of ions are also replaced by their corresponding bulk values ($\epsilon \approx \epsilon_S$, $u_\pm \approx u_\pm^b$). The electrostatic chemical potential of protein in Phase D can thus be

simplified to

$$\mu_P^{elec}(D) \approx Nv_0(u_+^b c_+^b + u_-^b c_-^b)\left(\frac{\epsilon_S - \epsilon_P}{\epsilon_S}\right) \tag{S18}$$

Combining Eqs. (S17) and (S18) together with the form of Born energy (Eq. (3) in the main text), the difference between $\mu_P^{elec}(D)$ and $\mu_P^{elec}(C)$ can be expressed as

$$\begin{aligned}\Delta\mu_P^{elec} &= \mu_P^{elec}(D) - \mu_P^{elec}(C)\\ &\approx Nv_0(u_+^b c_+^b + u_-^b c_-^b)\left(\frac{\epsilon_S - \epsilon_P}{\epsilon_S}\right) - Nv_0(c_+^b + c_-^b)\\ &= (z_+ + z_-)Nv_0 c_b\left[\frac{l_{B,S}}{2}\left(\frac{\epsilon_S - \epsilon_P}{\epsilon_S}\right)\frac{1}{\bar{a}} - 1\right]\end{aligned} \tag{S19}$$

where $c_\pm^b = z_\mp c_b$, $\bar{a}$ is the valency-weighted harmonic average radius of cation and anion given by $(z_+ + z_-)/\bar{a} = z_+^2 z_-/a_+ + z_-^2 z_+/a_-$, and $l_{B,S} = e^2/(4\pi\epsilon_0\epsilon_S kT)$ is the Bjerrum length in solvent.

IV. Supplementary figure

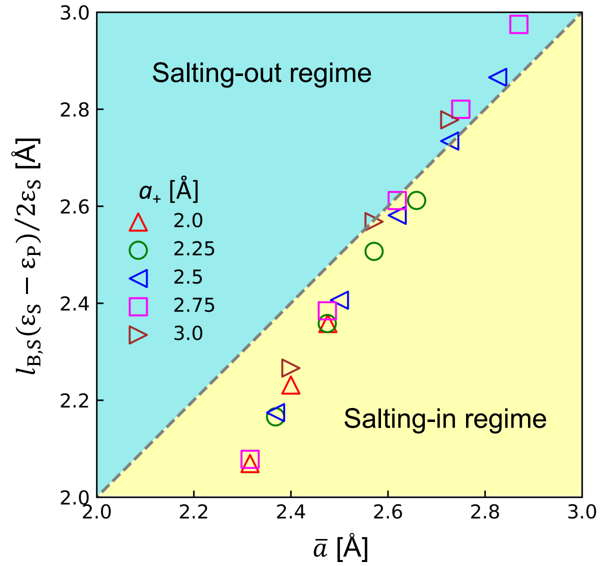

Figure S1: Comparison of the solubility behavior predicted by the analytical criterion (Eq. 11 in the main text) with the numerical calculation. Monovalent salt ions in water at $T = 298$K are considered ($z_+ = z_- = 1$, $\epsilon_S = 80$, and $l_{B,S} = 7.1\text{Å}$). Born radii of cations and anions are considered in the range of $2.0 \sim 3.0\text{Å}$, which covers most commonly used ions in experiments.[S4] $\bar{a}$ is the valency-weighted harmonic average radius $(z_+ + z_-)/\bar{a} = z_+^2 z_-/a_+ + z_-^2 z_+/a_-$. For a given value of $\bar{a}$, the critical ϵ_P is obtained from the numerical calculation of the turning point in the solubility curves (see Fig. 2 in the main text). The open symbols indicate the numerical results. The dash diagonal line is the analytical result of the universal criterion given by Eq. 11 for determining the boundary between the salting-in and salting-out regimes. The analytical result is in good agreement with the numerical calculations particularly for larger ions.

References

(S1) Fredrickson, G. H. *The Equilibrium Theory of Inhomogeneous Polymers* (Oxford University Press, New York, 2006).

(S2) Wang, Z.-G. Fluctuation in Electrolyte Solutions: The Self Energy. *Phys. Rev. E* **2010**, *81*, 021501.

(S3) Hoffman, J. D. *Numerical Methods for Engineers and Scientists* (Marcel Dekker, Inc., New York, 2001).

(S4) dos Santos A. P., Diehl, A. & Levin, Y. Surface Tensions, Surface Potentials, and the Hofmeister Series of Electrolyte Solutions. *Langmuir* **2010**, *26*, 10778-10783.
